# DIC-Aided Mechanoluminescent Film Sensor for Quantitative Measurement of Full-Field Strain

**DOI:** 10.3390/s25196018

**Published:** 2025-10-01

**Authors:** Guoqing Gu, Liya Dai, Liyun Chen

**Affiliations:** 1 School of Civil Engineering, Yancheng Institute of Technology, Yancheng 224051, China; yalidai1006@163.com; 2 Yancheng Institute of Supervision & Inspection of Product Quality, Yancheng 224056, China; ycszjzx103@163.cm; 3 Laboratory of Quality and Safety of Offshore Wind Power Equipment for Jiangsu Province Market Regulation, Yancheng 224056, China

**Keywords:** mechanoluminescent, film sensor, digital image correlation, strain quantitative measurement, calibration model

## Abstract

To break through the bottleneck in the mapping of the mechanoluminescent (ML) intensity field to the strain field, a quantification method for full-field strain measurement based on pixel-level data fusion is proposed, integrating ML imaging with digital image correlation (DIC) to achieve precise reconstruction of the strain field. Experiments are conducted using aluminum alloy specimens coated with ML film sensor on their surfaces. During the tensile process, ML images of the films and speckle images of the specimen backsides are simultaneously acquired. Combined with DIC technology, high-precision full-field strain distributions are obtained. Through spatial registration and region matching algorithms, a quantitative calibration model between ML intensity and DIC strain is established. The research results indicate that the ML intensity and DIC strain exhibit a significant linear correlation (R^2^ = 0.92). To verify the universality of the model, aluminum alloy notched specimen tests show that the reconstructed strain field is in good agreement with the DIC and finite element analysis results, with an average relative error of 0.23%. This method enables full-field, non-contact conversion of ML signals into strain distributions with high spatial resolution, providing a quantitative basis for studying ML response mechanisms under complex loading.

## 1. Introduction

In critical fields such as aerospace, automotive engineering, and large-scale civil infrastructure, structural components are subjected to complex dynamic loads under multi-field coupled environments throughout their service life. The reliability of their mechanical performance not only directly affects the overall operational efficiency of equipment systems but also plays a vital role in ensuring personnel safety. Therefore, the accurate and real-time monitoring of the stress state and deformation behavior of key components holds significant engineering value for preventing structural failure and extending service life. Among them, strain, as a key mechanical parameter characterizing the local deformation and damage evolution of materials [1], has become an indispensable technical indicator in structural health monitoring (SHM) systems for its precise measurement.

Current strain measurement techniques are mainly categorized into contact and non-contact methods [1]. Contact methods include strain gauges [2], fiber Bragg grating (FBG) sensors [3,4], and piezoelectric sensors [5,6]. Although strain gauges are small in size and low in cost, they provide only point measurement data and are susceptible to temperature-induced drift. FBG sensors have the advantages of anti-electromagnetic interference and multi-point series measurement, but they are relatively expensive and rely on professional demodulation equipment. Among non-contact methods, digital image correlation (DIC) [7] has emerged as a widely adopted optical technique. It reconstructs full-field strain by tracking the grayscale distribution of random speckle patterns. Due to its high measurement accuracy and flexible experimental arrangement, DIC has been increasingly applied in the field of SHM. The research focus has transferred from controlled laboratory environments to long-term monitoring of real, large-scale infrastructure, such as long-span bridges [8], wind turbine blades [9,10], and historic buildings [11]. However, DIC also has certain limitations, including sensitivity to outdoor lighting, weather, and vibration conditions, and reliance on computationally intensive image processing algorithms.

To overcome the limitations of the aforementioned strain-sensing techniques, a novel optical detection method based on mechanoluminescent (ML) materials [12,13] has become a research hotspot in the field of direct strain visualization. Compared with DIC, ML measurement has the advantages of high spatial resolution, passive operation, and strong environmental adaptability. In the field of SHM, sensing technologies based on ML have demonstrated significant application potential, such as in impact damage detection of composite materials [14,15] and the dynamic visualization of crack propagation [16,17]. Notably, recent research highlights a key trend: integrating ML optical sensing with machine learning algorithms to develop next-generation intelligent SHM systems. For instance, existing studies have proposed hybrid architectures that integrate full-field ML information sensing, deep learning-based data analysis, and finite element simulation [18]. This approach enables multimodal sensing, automated damage diagnosis, and real-time validation of structural conditions.

Despite the remarkable progress in qualitative analysis, the quantitative application of ML technology still faces several challenges. First, photoluminescence (PL) afterglow induced by ultraviolet (UV) excitation can interfere with the strain signal. This issue can be addressed either by maintaining a stable PL background through continuous UV illumination [19,20] or by allowing the sample to rest for several minutes under stress-free conditions after ceasing UV illumination to allow the afterglow to naturally decay, thereby obtaining a relatively constant PL background [21,22]. Second, how to achieve precise calibration of the strain field while maintaining the luminescent properties of ML remains a core issue that urgently needs to be addressed.

Currently, two complementary solutions have emerged in this field. The first method involves integrating ML films onto the surface of the test specimen, utilizing its highly sensitive light intensity response characteristics to obtain full-field strain information, and combining finite element analysis (FEA) to establish a light intensity-strain mapping relationship [23,24]. The advantage of this method lies in its simple experimental setup, which enables non-contact measurement without additional optical markers, making it particularly suitable for strain monitoring of complex geometric components. However, since FEA simulations are based on idealized material constitutive relationships and boundary conditions, their computational results exhibit systematic deviations from the actual physical strain field, thereby limiting calibration accuracy to some extent. To overcome this limitation, the second method innovatively utilizes the luminescent particles in the ML film as a natural speckle field, directly calculating the true strain distribution on the specimen surface via DIC algorithm [19,24]. The prominent advantage of this technical approach lies in establishing calibration relationships entirely based on experimental measurement data, thereby avoiding uncertainties introduced by theoretical models. It should be noted that a basic assumption of the DIC algorithm is that speckle serves as a deformation marker, and the brightness remains constant or linearly changes. However, the brightness of ML particles is different before and after deformation compared to traditional DIC speckles, so that the normalized cross-correlation coefficient (NCC) of ML particles is significantly below the NCC precision threshold of 0.98–0.99 required by the traditional DIC algorithm. Additionally, this method is highly sensitive to the film fabrication process, with even minor variations in particle distribution uniformity or surface morphology potentially significantly affecting measurement reliability. Current research status indicates that both methods have their own advantages: the former offers the advantage of a simple system but is limited by model accuracy, while the latter can obtain true strain but faces challenges in measurement stability. Therefore, developing a new calibration method that combines theoretical rigor and experimental reliability remains an important research direction for advancing the engineering application of ML technology.

To overcome these challenges, this study introduces a dual-sided synchronous measurement methodology that spatially decouples the functions of DIC and ML sensing. In contrast to the integrated-film approach of Shin et al. [19], a high-contrast speckle pattern is applied on one side of the specimen for high-fidelity DIC strain measurement, while an ML film is coated on the opposite side to capture luminescent response under identical loading conditions. This configuration preserves the advantages of both techniques: the DIC side provides an accurate, model-free strain reference, while the ML side enables full-field, passive sensing. The establishment of the light intensity-strain mapping relation is, therefore, based on direct experimental correlation rather than numerical simulation, significantly improving reliability and traceability. The accuracy of this calibration is rigorously validated through tensile testing of notched specimens, with detailed precision analysis performed on the calibration curves. This method combines the high accuracy of DIC with the high resolution and passive characteristics of ML, potentially enabling a distributed, passive, and traceable strain measurement system that meets both the requirements for large-area online monitoring and local detail imaging.

## 2. Brief Principle of Three-Dimensional DIC

Three-dimensional DIC (3D-DIC) is a non-contact optical technique that combines binocular stereo vision with two-dimensional DIC (2D-DIC) to measure full-field surface deformations and strains. The method begins with the calibration of the binocular vision system to determine the intrinsic and extrinsic parameters of the left and right cameras. Once calibrated, the system captures a pair of reference images of the specimen surface before deformation. Subsequently, during mechanical loading or deformation, a series of deformed images is recorded synchronously by both cameras.

In the left reference image, a region of interest (ROI) is selected, and it is subdivided into small square subsets for analysis. Each subset serves as a tracking unit. Using 2D-DIC, the corresponding position of each subset in the right reference image is identified through correlation matching, enabled by extreme geometric constraints after stereo rectification. This initial matching, combined with the calibrated camera parameters, allows reconstruction of the 3D coordinates of surface points via triangulation, establishing the undeformed 3D coordinate field.

**Figure 1:**
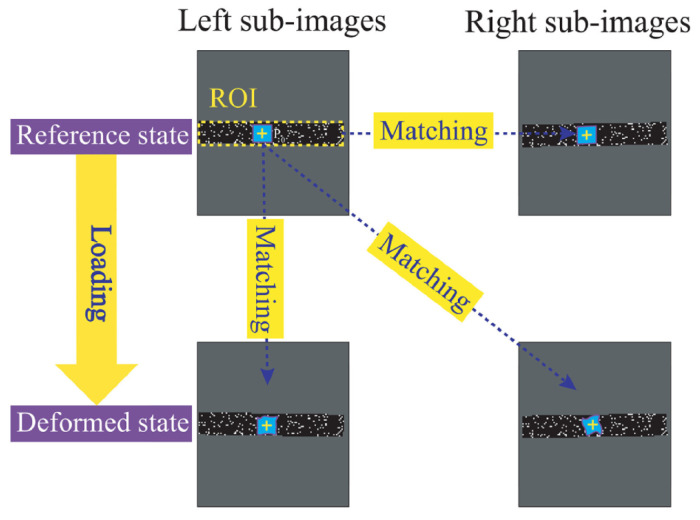
Three-dimensional DIC calculation schematic diagram.

As shown in Figure 1 [25], when deformation occurs, the same subsets in the left camera’s deformed images are matched using a correlation criterion—typically the normalized cross-correlation (NCC) function—to determine their new positions and, thus, the in-plane displacement components. Simultaneously, the corresponding subsets in the right camera’s deformed images are matched to maintain stereo correspondence. The updated image coordinates from both views are then used to reconstruct the deformed 3D positions of the points. By comparing the 3D coordinates before and after deformation, the full-field 3D displacement field is computed. Strain fields can subsequently be derived through spatial differentiation of the displacement data.

## 3. Experimental Tests

### 3.1. Fabrication of the ML Film Sensors

The green photon-emitting ML powder SrAl_2_O_4_: Eu^2+^, Dy^3+^ (SAOED) with excitation and emission peaks at 360 nm and 520 nm, respectively, was purchased from Shenzhen Zhanwanglong Technology Co., Ltd., Shenzhen, China. The epoxy resin (West System 105) and its curing agent (West System 206) were obtained from Gougeon Brothers, Bay City, MI, USA. Additionally, a 1.5 mm thick aluminum alloy plate (AA6061) was used as the substrate material in this work. Its relatively smooth surface and dimensional stability provide a reliable base for film adhesion applications, facilitating uniform dispersion and adhesion of the mechanoluminescent composite layer.

First, the ML powder, SAOED, and epoxy resin were weighed in a 1:1 mass ratio and thoroughly mixed to form a homogeneous solution. Next, the curing agent was added at a mass ratio of 5:1, and the mixture was stirred using a magnetic stirrer at a speed of 500 rpm for 15 min to ensure that all components were uniformly dispersed in the solution. Then, the well-mixed composite solution was slowly poured into a customized polytetrafluoroethylene (PTFE) mold with a fixed internal cavity depth of 0.4 mm, and excess solution was gently removed along the edges using a scraper to ensure uniform film thickness. Finally, the mold was placed in an oven and cured at 80 °C for 1 h, forming an ML film with a thickness of approximately 0.4 mm.

### 3.2. Preparation of Specimens for ML Films Adhesion

**Figure 2:**
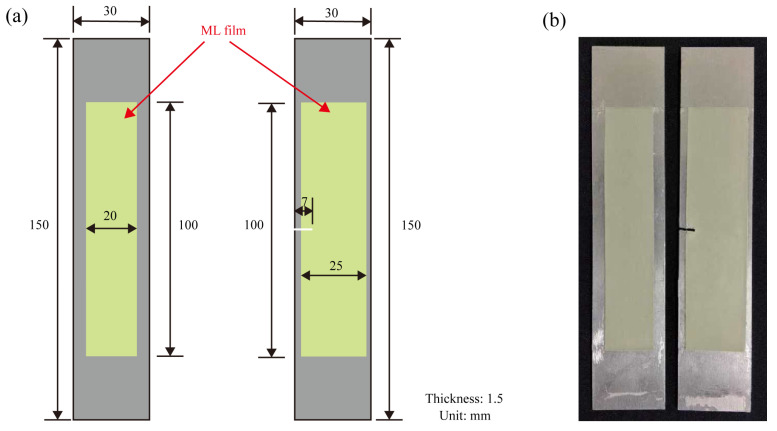
The prepared specimens’ schematic diagram and their physical picture. (**a**) Dimensions of the uniaxial tension specimens without and with a notch; (**b**) Physical specimens’ picture.

Here, the aluminum alloy thin plate specimens without and with a notch were elaborately prepared. To ensure strong adhesion between the ML film and the specimens’ surface, surface pretreatment of the specimens was carried out. The aluminum alloy surface was first polished to increase surface roughness and enhance mechanical adhesion, followed by thorough cleaning of the specimens’ surface with alcohol and acetone to remove any residual oil, dust, or other impurities. After cleaning and drying, the ML film was adhered to the specimens’ surface using transparent epoxy adhesive and cured at room temperature for 12 h, thereby completing the specimens’ preparation. Meanwhile, the high-quality artificial speckle patterns were carefully painted using commercial black and white paints on the backside of the well-adhered ML film specimens. The dimensions of the specimen and film are shown in Figure 2.

### 3.3. Testing Procedures

**Figure 3:**
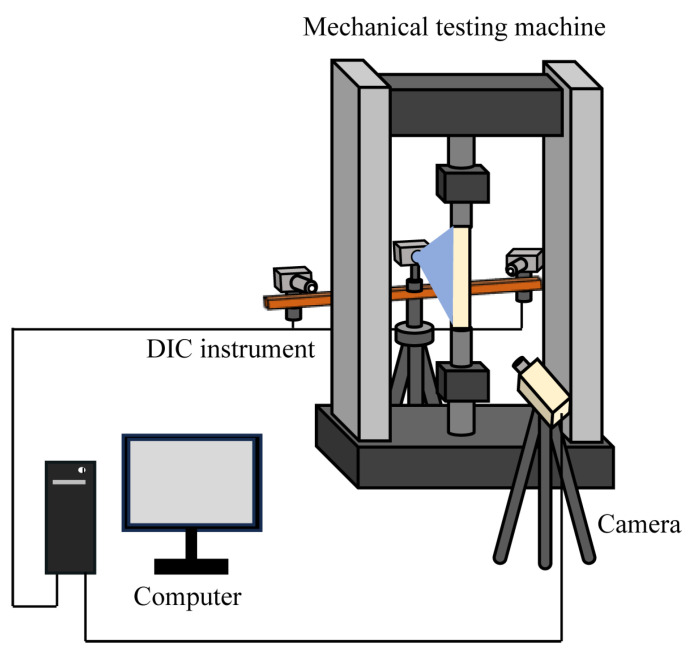
Schematic diagram of the experimental test setup.

The prepared specimens shown in Figure 3 were installed in the universal testing machine’s dedicated tensile fixture, ensuring that the specimen was axially centered (see Table 1 for relevant parameters of the testing machine). The specimen was stretched in a unidirectional way with the bottom fixed and the top stretched, and under a constant loading rate of 6 mm/min. Prior to testing, the ML film was continuously excited for 2 min using a 10 W UV light source (wavelength range: 350–450 nm) to achieve saturated luminescence. Subsequently, it was aged for 3 min in an unloaded state to reduce the impact of stressless PL attenuation on the ML signal. Then, the uniaxial tension experiments of two types of specimens (without and with a notch) were successively implemented.

During the tensile test, the FLIR GS3 CCD camera (FLIR Systems, Inc., Wilsonville, OR, USA, Resolution: 2448 pixel × 2048 pixel) and Fujinon HF16SA-1 len (see Table 2 for relevant parameters of the len) captured ML emission images at a speed of 5 fps, with the camera’s optical axis pointing vertically toward the ML film surface and a fixed distance of 25 cm maintained between the lens and the film surface. Simultaneously, full-field strain distributions on the specimen’s backside surface were monitored in real time at the same sampling rate using the 3D-DIC system (Hefei Zhongke Junda Vision Technology Co., Ltd., Hefei, China), ensuring temporal consistency between the ML signal and the strain data. The experiment employed an optimized design with front and back separation: the front surface of the specimen was adhered with an ML film for optical detection, while the back surface was sprayed with a random speckle pattern for DIC measurement.

A light-blocking isolation device was installed in the middle of the testing machine. This design addresses the dual issues of speckle interference affecting ML signals and DIC light sources impacting ML image acquisition in traditional same-side measurements. Throughout the experiment, a light shield was used to isolate ambient light and ensure the accuracy of data collection. Figure 3 shows the overall experimental setup, including the spatial arrangement of the loading device, optical detection unit, and data acquisition systems.

## 4. Methods

### 4.1. Data Extraction

During the acquisition of ML and DIC images, the use of different imaging systems—industrial cameras for ML and specialized instruments for 3D-DIC—resulted in inconsistent image coordinate systems, preventing direct spatial registration. To ensure accurate correlation in the light intensity-strain relationship, data points from both datasets had to correspond to the same physical location on the specimen. To achieve this, a physical scale calibration was implemented by marking the four corner points of a predefined ROI directly on both front and back surfaces of the specimen. These physical markers defined an identical ROI area visible to both imaging systems. By referencing these shared spatial landmarks, image coordinates from both ML and DIC were converted into a common physical coordinate system, enabling precise spatial alignment, cross-system data matching, and reliable joint analysis.

### 4.2. Image Processing

**Figure 4:**
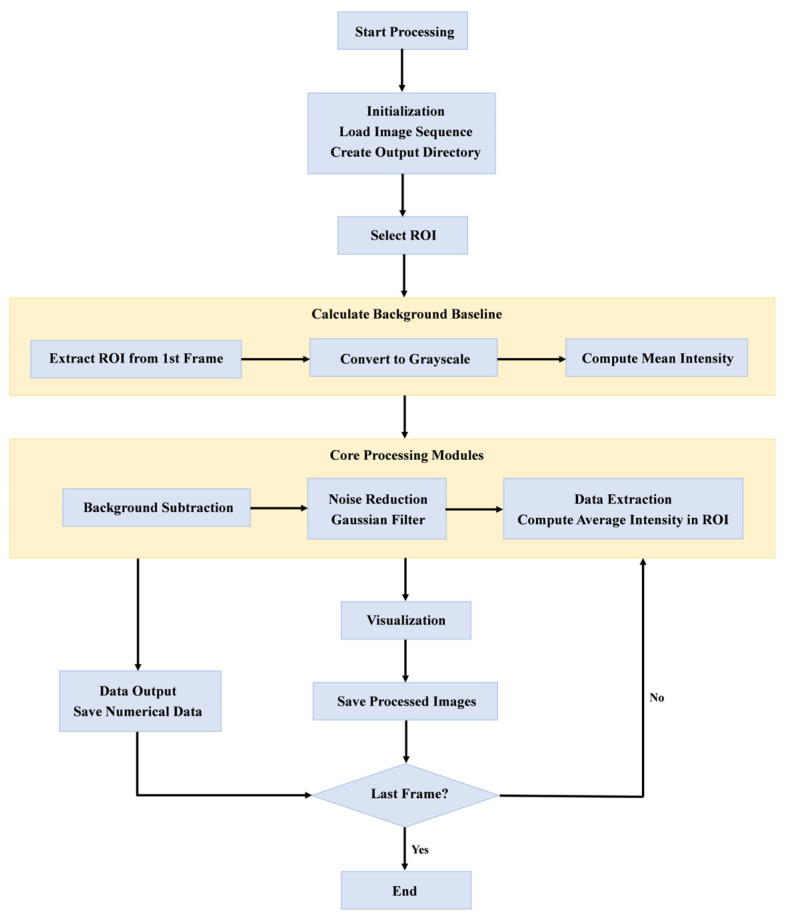
Image processing algorithm flow chart.

To quantitatively characterize the evolution of the ML response during the tensile process of aluminum alloy specimens, image processing and analysis were performed using custom Python scripts (Python Software Foundation, version 3.12). The algorithm mainly implemented the following three core processing steps, with its overall workflow illustrated in Figure 4:

(1) Background average light intensity calculation: since the ML film exhibited a certain degree of self-luminescence when exposed to UV light without loading, background light intensity had to be corrected to ensure data accuracy. The algorithm first extracted the grayscale image of the specified ROI area from the initial frame image and calculated the average grayscale value within that area as the background average light intensity ${\bar{I}}_{bg}$, which was used for background subtraction in subsequent images.
(2) Background subtraction: for each frame of the image, its grayscale matrix within the same ROI range was extracted, and background subtraction processing was performed according to the following formula:
  (1) $$I^{*}\left(x,y\right)=\max\left[I_{raw}\left(x,y\right)-{\bar{I}}_{bg},0\right]$$

The resulting image data retained only the luminescence enhancement caused by mechanical loading.

(3) Visualization: each frame of background-corrected image data was visualized in the form of a false-color image, facilitating analysis of the distribution of light intensity and its evolution with load.

Strain data was directly calculated using a commercial 3D-DIC software (RDIC-STD-DH1200, Hefei Zhongke Junda Vision Technology Co., Ltd., Hefei, China) and served as reference information for ML image data, which was used to establish and analyze the subsequent light intensity-strain mapping relationship.

### 4.3. Numerical Modeling

To gain deeper insight into the stress concentration phenomenon around the defect and to provide a theoretical basis for interpreting the experimental ML response, a finite element model was established using the commercial software ABAQUS (version 2024). The geometric model replicated the dimensions of the notched 6061 aluminum alloy specimen. The material was modeled as an isotropic, linear elastic solid, with properties including Young’s modulus and Poisson’s ratio provided in Table 3.

The model was meshed using structured hexahedral elements (C3D8R), which help avoid mesh distortion issues near the notch and ensure computational accuracy in the stress concentration region. A suitable global seed size was defined, with local refinement applied around the notch to capture stress gradients effectively.

The response of this discretized system is governed by the fundamental principles of linear elastic statics. From a computational mechanics perspective, the mechanical behavior of the specimen is governed by the classical equations of linear elasticity. The fundamental system consists of the equilibrium equation, the strain-displacement relation, and the constitutive law. Specifically, the infinitesimal strain tensor is defined as $\varepsilon_{ij}=\frac{1}{2}(\frac{\partial u_{i}}{\partial x_{j}}+\frac{\partial u_{j}}{\partial x_{i}})$, and the stress–strain relationship follows Hooke’s law for isotropic materials [26]:

(2) $$\sigma_{ij}=\lambda \varepsilon_{kk}\delta_{ij}+2\mu \varepsilon_{ij}$$

where *λ* and *μ* are the Lamé constants, related to Young’s modulus *E* and Poisson’s ratio *ν* by $\lambda =\frac{Ev}{\left(1+v\right)\left(1-2v\right)}$ and $\mu =\frac{E}{2\left(1+v\right)}$. The governing equation is solved subject to appropriate boundary conditions, including prescribed displacements and traction loads.

Within this theoretical framework, a static general analysis step was employed to simulate the quasi-static tensile process. The boundary conditions replicated the experimental setup: one end of the specimen was fully fixed, while a prescribed displacement of 6 mm—matching the final displacement achieved in the physical experiment—was applied to the opposite end along the axial direction.

The primary objective of the simulation was to compute the full-field strain distributions for quantitative correlation with the ML intensity patterns. It should be noted that the current model assumes linear elasticity and does not account for plasticity or damage at higher strain levels.

Therefore, it is important to acknowledge the limitations of the current finite element model. Firstly, the material was modeled as perfectly linear elastic, which does not capture the potential plastic deformation or damage initiation that may occur in the aluminum alloy at higher stress levels near the notch. Secondly, the model assumed a perfectly bonded interface between the ML film and the substrate, neglecting the potential influence of interfacial shear or debonding effects on strain transfer that may occur during actual experiments. Lastly, the simulations were conducted under quasi-static conditions and did not account for any strain-rate-dependent material behavior. These simplifications were necessary to establish a baseline understanding but suggest avenues for future work.

## 5. Experimental Results and Discussions

### 5.1. Quantitative Calibration Relationship Between ML Intensity and DIC Strain

**Figure 5:**
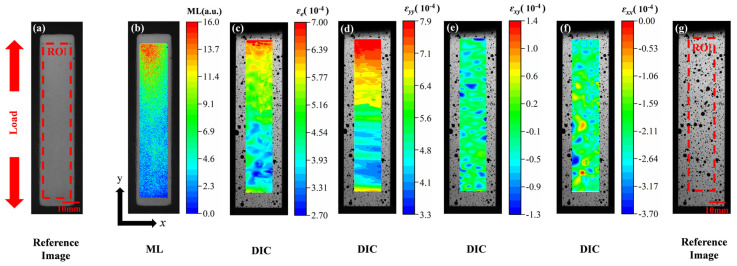
Comparison results between ML and strain cloud maps. (**a**) Reference image of ML film; (**b**) ML cloud map; (**c**) Von Mises effective strain field based on DIC; (**d**) Strain field in the *y*-direction (*ε_yy_*) measured by DIC; (**e**) Shear strain field (*ε_xy_*) measured by DIC; (**f**) Strain field in the *x*-direction (*ε_xx_*) measured by DIC; (**g**) Reference image for DIC strain measurement.

This section conducted a comparative analysis of the deformation behavior of aluminum alloy specimens during the elastic stage by simultaneously collecting ML images and DIC strain maps. As shown in Figure 5, the reference images of the ML film (Figure 5a) and the DIC reference image (Figure 5g) both have the same ROI (90 × 16 mm) and physical scale (10 mm) marked. Figure 5b shows the ML image of the elastic stage and its intensity cloud map generated by Python. The light intensity exhibited a significant non-symmetric distribution along the axial direction of the specimen: the intensity was highest in the upper region, gradually decreasing downward, with the signal in the lower clamping zone significantly reduced to near extinction. This distribution was highly consistent with the mechanical boundary conditions: during tensile testing, the upper end of the specimen underwent controlled displacement while the lower end was fixed, resulting in a gradient distribution of the strain field. The maximum strain was located near the upper free end, while the lower constrained region exhibited lower strain levels. The spatial variation in ML intensity directly reflected the strain gradient characteristics, indicating its high sensitivity to local stress/strain states.

The 3D-DIC system simultaneously acquired the multi-dimensional strain fields on the back of the specimen, as shown in Figure 5c (*ε_e_*), Figure 5d (*ε_yy_*), Figure 5f (*ε_xx_*), and Figure 5e (*ε_xy_*). Figure 5d shows that the maximum value of the *y*-direction strain (*ε_yy_*) (approximately 7.9 × 10^−4^) was concentrated at the top of the specimen and decreased downward, with its distribution highly consistent with the ML cloud map. In Figure 5f, the *x*-direction strain (*ε_xx_*) exhibited a negative (compression) state overall, indicating that the transverse contraction conforms to the Poisson effect. In Figure 5e, the shear strain had a low amplitude and uniform distribution, indicating that no significant eccentricity or torsion occurred during loading, ensuring the ideal nature of the uniaxial tensile state. Of particular importance, the von Mises effective strain (*ε_e_*) cloud map shown in Figure 5c comprehensively reflects the coupled effects of multiaxial strain, exhibiting a high degree of consistency with the ML intensity field in terms of spatial distribution: the maximum effective strain was located at the upper part of the specimen and decreased gradually downward, almost perfectly corresponding to the trend of light intensity. This indicates that the ML response is not solely dependent on a single strain component but may be related to the overall deformation energy density or equivalent strain level. Therefore, the ML intensity distribution can be used as an optical mapping of the effective strain field, providing direct visual evidence for characterizing the deformation behavior of materials.

**Figure 6:**
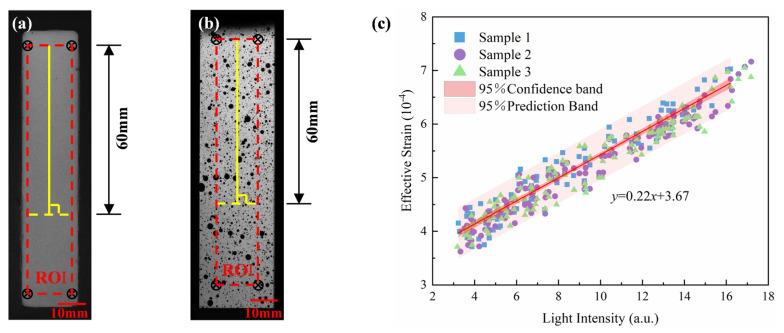
Data extraction and linear fitting of ML intensity and strain distribution. (**a**) Reference image of ML intensity data extraction; (**b**) Reference image of DIC strain data extraction; (**c**) Light intensity-strain linear fitting results.

To further establish a quantitative relationship between light intensity and strain, light intensity and strain data were extracted from the upper two-thirds of the region along the vertical centerline of the specimen (height 60 mm, corresponding to the longitudinal range of the ROI) (Figure 6a,b), and a linear regression analysis was performed on the combined dataset from three independent tests. The results indicated a significant linear relationship between light intensity (*x*, units: a.u.) and strain (*y*, × 10^−4^) (Figure 6c), with the fitting equation being:

(3) y=0.22x+3.67

The quality of the fit was excellent, as evidenced by a coefficient of determination (R^2^) of 0.922. The 95% confidence and prediction bands accompanying the regression line in Figure 6c further confirm the statistical robustness of this calibration relationship. Regarding the spatial resolution of the system, the proposed ML-based method demonstrates a detectable strain gradient of approximately 4.11 × 10^−4^. The fitted slope (0.22) reflects the strain increment corresponding to the change in unit light intensity and can be regarded as the stress-optical response coefficient of the material-film system, which has potential calibration and application value. It is worth noting that the intercept (3.67) of the fitted curve is not caused by background noise or the intrinsic luminescence of the film. The physical origin of this intercept lies in the inherent characteristics of the strain field of the specimen under elastic tensile stress. Under the condition of axial tension, the lower end of the specimen was fixed while the upper end was subjected to tensile loading, resulting in a continuous gradient distribution of strain along the axial direction. Despite the weak or even negligible ML response in the lower region near the clamping end, DIC could still detect non-zero micro-strain values (Figure 5c). This baseline strain reflects the continuity of the specimen’s overall elastic deformation, consistent with classical elasticity theory: under uniform tensile loading, the strain field is continuously distributed in space, and there is no absolute zero-strain region. Therefore, even when light intensity approaches zero, strain still has a non-zero minimum value, manifested as the positive intercept of the fitted straight line. This phenomenon further illustrates that the ML signal primarily responds to local stress changes, while DIC measurements capture the global continuous deformation field. The two complement each other in their physical response mechanisms, jointly revealing the multi-scale characteristics of material deformation.

### 5.2. Verification of ML Technology by Quantitative Measurement of Notch-Tip Strain

**Figure 7:**
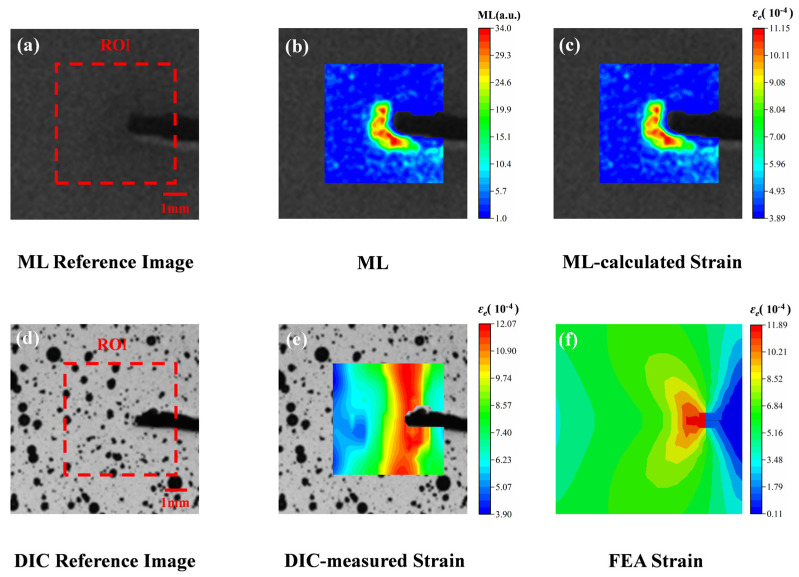
Comparison of strain distribution across the entire field of notch specimens characterized by three methods. (**a**) ML reference image (including ROI calibration); (**b**) Elastic stage ML cloud map; (**c**) Calculation of strain field based on light intensity-strain relationship; (**d**) DIC speckle reference image; (**e**) DIC measured effective strain field distribution; (**f**) Theoretical strain field by FEA.

In previous studies, a linear calibration relationship between light intensity and strain was established through axial tensile tests on complete specimens. To further validate the applicability of this relationship under different stress conditions, aluminum alloy specimens with artificial notches (7 mm × 2 mm) were specifically designed for axial tensile tests. Figure 7 systematically displays the various image data obtained during the experiment. Figure 7a shows the reference image of the film after optical excitation before the sample was stretched, clearly marking the ROI region and a 1 mm physical scale. At the elastic deformation stage, luminescence images of the film were captured (Figure 7b) and subsequently processed through a Python program to produce ML cloud maps. Based on the light intensity data of each pixel point in the ROI region of Figure 7b, the strain values at each point were calculated using the previously established calibration relationship (Equation (3)), and the strain visualization cloud map of the sample was finally plotted (Figure 7c). For comparison and verification, the sample surface with a sprayed speckle pattern was imaged for DIC reference (Figure 7d), and the effective strain distribution was calculated using 3D-DIC software (RDIC-STD-DH1200, Hefei Zhongke Junda Vision Technology Co., Ltd., Hefei, China) (Figure 7e). Additionally, the theoretical strain distribution near the notch on the surface was obtained through FEA (Figure 7f).

A comparative analysis of these three cloud maps (Figure 7c,e,f) reveals a high degree of consistency in terms of strain distribution trends and numerical ranges. In particular, in the region near the tip of the notch, all three methods clearly show strain concentration phenomena. This consistency strongly validates the reliability of the previously established light intensity-strain relationship (Equation (3)) under different stress conditions.

To critically validate the experimental observations and gain deeper insights into the stress state, the finite element model described in Section 4.3 was employed. Figure 7e,f presents a comparative view of the von Mises stress distribution obtained from FEA and the strain field derived from DIC at the same load level. The high-stress zone in the simulation closely coincides with the high-strain region measured by DIC, providing qualitative validation of the model’s accuracy.

**Figure 8:**
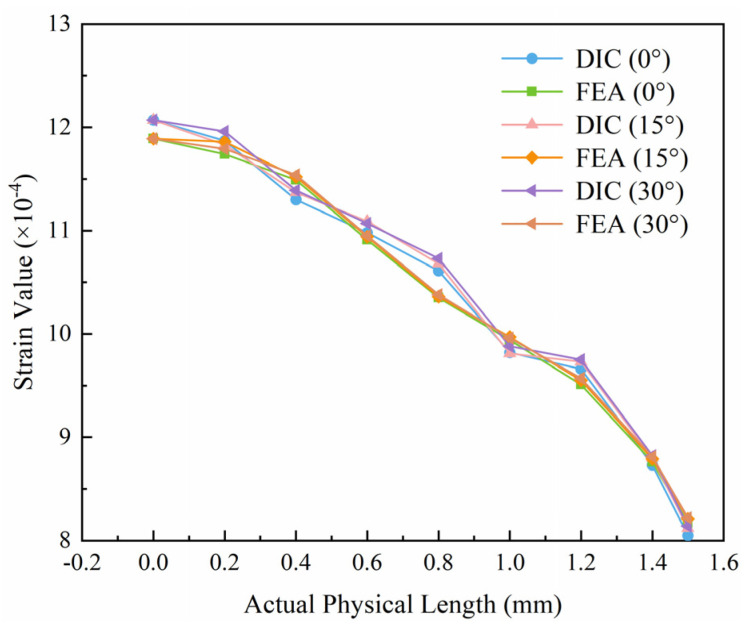
Comparison of strain values between DIC and FEA.

For quantitative analysis, strain values were extracted from FEA and DIC strain maps at 0°, 15°, and 30° orientations along a 1.5 mm length near the notch tip for comparison. As shown in Figure 8, the strain values from both methods exhibit excellent consistency. The maximum strain at the notch root in FEA is 1.189 × 10^−3^, differing from the DIC measurement 1.207 × 10^−3^ by only 0.18 × 10^−3^. This minor discrepancy can be attributed to the idealized boundary conditions in the simulation failing to fully replicate the complex clamping effects present in the experiment.

**Figure 9:**
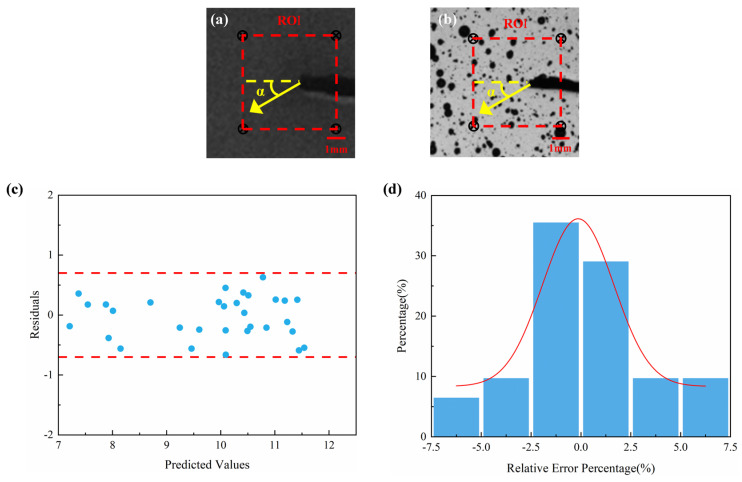
Data extraction and error analysis between ML and DIC. (**a**) Schematic diagram of ML-calculated strain value data extraction; (**b**) Reference image of DIC strain data extraction; (**c**) Residual plot; (**d**) Relative error distribution histogram.

To further evaluate measurement accuracy, a sector area was defined from the tip of the notch in the direction ranges of 10° to 20°, and data from a 1 mm path within that area were extracted for analysis. As shown in Figure 9, the residuals between ML and DIC are generally distributed around zero, indicating good consistency between the calculated strain values and the actual strain values. Furthermore, most of the residual values are concentrated within the range of [−0.7, 0.7], indicating that the deviation between the calculated strain values and the actual strain values is small. Meanwhile, relative error histogram analysis shows that most data points have relative errors within ±2.5%, indicating that the error between the calculated strain values and the actual strain values is small, with high precision. The average relative error is approximately 0.23%, and the standard deviation is approximately 3.53%, further validating the reliability of the ML-calculated strain values.

Compared to the existing integrated ML-DIC method proposed by Shin et al. [15], our dual-sided configuration provides a more reliable foundation for quantitative analysis. While their approach innovatively employs ML films as speckle patterns, inherent uncertainties in DIC accuracy arise from variations in speckle brightness. Our approach achieves calibration coefficient determination with explicit statistical confidence by decoupling sensing mechanisms, enhancing measurement reliability and repeatability. This provides a more effective solution for quantitative strain analysis under complex operating conditions.

## 6. Conclusions

This study successfully achieved non-contact, full-field visualization and quantitative monitoring of the stress–strain state of aluminum alloy specimens under elastic loading conditions by combining ML film sensors with DIC. The experiment employed a dual-sided synchronous measurement scheme, with ML films adhered to the front surface of the specimen and a speckle field prepared on the back surface. An industrial camera and DIC system were used to separately capture ML images and surface deformation images, ensuring spatiotemporal consistency. By spatially aligning and region-matching the light intensity during the elastic stage with the strain field measured by DIC, a pixel-level data-based light intensity-strain calibration relationship was established. The results showed a significant linear correlation between the two within the studied range.

The calibration relationship was further validated on specimens with an artificial notch defect. By substituting the light intensity data from the ML images into the established linear model, the calculated strain cloud map was reconstructed. Its spatial distribution trend highly matched the DIC-measured strain field and finite element simulation results, particularly maintaining good morphological consistency in the high-strain concentrated regions near the notch tip, verifying that the method retains high quantitative accuracy even in local high-strain regions.

Quantitatively, the method demonstrated high accuracy and reliability. The calibration yielded a strong linear relationship (R^2^ = 0.92) between ML intensity and strain. Residual analysis confirmed that most errors were confined within ±0.7, while relative errors were predominantly within ±2.5%, with a mean relative error of 0.23% and a standard deviation of 3.53%. These quantitative metrics affirm the robustness of the proposed approach for strain field reconstruction.

This method establishes a conversion pathway from the ML intensity field to the physical strain field while retaining the spatial resolution of the original image, thereby facilitating a transition of ML technology from qualitative observation toward quantitative characterization. The operational compatibility between ML films and DIC algorithms in data acquisition and processing suggests that the approach can be adapted to various ML material systems. However, it is important to note that the present validation is confined to static elastic loading under well-controlled laboratory conditions.

To improve the credibility and mechanistic depth of the methodology, future work will focus on: (1) extending the technique to nonlinear and plastic deformation regimes to establish a unified elastoplastic constitutive model correlating luminescence and strain; (2) integrating multi-modal measurement techniques, including Raman spectroscopy for local crystal structure and strain analysis, XPS mapping for chemical state evolution under stress, and high-speed imaging for transient luminescence dynamics. Such synergistic validation will significantly enhance the interpretation of ML mechanisms and strengthen the reliability of strain field reconstruction under complex mechanical conditions; (3) testing the method in real structural environments to evaluate its practicality and limitations beyond idealized settings.

## Figures and Tables

**Table 1:** Relevant parameters of the universal testing machine.

Technical Parameter	Parameter Value
Brand	Meters (MTS)
Model	E 43.504
Manufacturer	Meters Industrial Systems Co., Ltd. (Shanghai, China)
Range	20 N–50 KN
Deformation measurement range	0.2–100%FS
Relative error of deformation indication	Within ±0.5%
Relative error of displacement indication	Within ±0.5%
Relative error of test force indication	±0.5%

**Table 2:** Relevant parameters of the camera lens.

Technical Parameter	Parameter Value
Brand	Fujinon
Model	HF16SA-1
Manufacturer	Fujifilm Co., Ltd. (Tokyo, Japan)
Focus type	Fixed focal length
Focal length	16 mm
Resolution	5 M
Aperture range	F/1.4–F/22

**Table 3:** Material properties of simulation experiment.

Parameter	Value
Density/(kg·m^−3^)	2700
Young’s modulus/Gpa	68
Poisson’s ratio	0.33
Maximum principal stress/Mpa	84
Damage viscosity coefficient	0.0004

## Data Availability

The raw data supporting the conclusions of this article will be made available by the corresponding author on request.
